# Mutual age-varying influences of binge drinking and cannabis use during emerging adulthood in the NCANDA cohort

**DOI:** 10.1111/acer.70139

**Published:** 2025-09-29

**Authors:** Jack T. Waddell, Ty Brumback, Fiona C. Baker, Shayna Cheek, Duncan B. Clark, David B. Goldston, Jeremy L. Grove, Bonnie J. Nagel, Kate B. Nooner, Adolf Pfefferbaum, Kilian M. Pohl, Edith V. Sullivan, Susan F. Tapert, Wesley K. Thompson, Sandra A. Brown

**Affiliations:** 1 University of California San Diego, La Jolla, California, USA; 2 Xavier University, Cincinnati, Ohio, USA; 3 SRI International, Menlo Park, California, USA; 4 Duke University, Durham, North Carolina, USA; 5 University of Pittsburgh, Pittsburgh, Pennsylvania, USA; 6 Oregon Health & Science University, Portland, Oregon, USA; 7 University of North Carolina Wilmington, Wilmington, North Carolina, USA; 8 Stanford University School of Medicine, Stanford, California, USA; 9 Laureate Institute for Brain Research, Tulsa, Oklahoma, USA

**Keywords:** binge drinking, cannabis, emerging adulthood, marijuana

## Abstract

**Background:**

Binge drinking peaks during emerging adulthood and is associated with negative developmental outcomes. Within-person changes in cannabis use have been shown to coincide with binge drinking; however, whether within-person changes in binge drinking and cannabis use prospectively predict one another and whether these relations vary by age remain unknown. The current study sought to fill these gaps.

**Methods:**

Data come from National Consortium on Alcohol and Neurodevelopment (NCANDA) participants aged 18–25 years reporting alcohol and cannabis use (*N* = 526). Parallel-process state–trait mixed effect growth models tested whether: (1) binge drinking across emerging adulthood was correlated with cannabis use (random intercepts); (2) steeper growth in binge drinking across emerging adulthood correlated with growth in cannabis use (random slopes); and (3) age-specific, within-person changes in binge drinking/cannabis use reciprocally predicted one another.

**Results:**

Across individuals, more frequent binge drinking was correlated with more frequent concurrent cannabis use, and steeper increases in binge drinking were correlated with steeper increases in cannabis use during emerging adulthood. Within-person changes in binge drinking and cannabis use covaried. Within-person increases in cannabis use predicted subsequent increases in binge drinking between ages 18 and 21 years but decreases in binge drinking between ages 24 and 25 years. Within-person changes in binge drinking did not predict subsequent changes in cannabis use during emerging adulthood.

**Conclusions:**

Changes in cannabis use coincided with changes in binge drinking, concurrently and subsequently, particularly between ages 18 and 21 years when changes in cannabis use predicted subsequent increases in binge drinking and ages 24 and 25 years when changes in cannabis use predicted decreases in subsequent binge drinking. Incorporating motivational approaches to reduce cannabis use in alcohol interventions may be efficacious in early emerging adulthood.

## INTRODUCTION

Binge drinking (i.e., drinking 4+ drinks on an occasion for females, 5+ for males assigned at birth) remains a serious public health concern. Rates of binge drinking remain steadily high in recent decades (Grant et al., 2017; McKetta et al., 2021), and binge drinking is associated with negative consequences, including risky sexual behavior, academic/occupational dysfunction, and more impaired driving infractions (e.g., Courtney & Polich, 2009; Evans-Polce et al., 2022; Perkins, 2002; Waddell et al., 2021; Weschler et al., 1994; Wycoff et al., 2025). Binge drinking creates an estimated economic burden of $250 billion yearly (Manthey et al., 2021), making it crucial to expand our understanding of factors to enable the development of effective prevention and early intervention efforts.

Binge drinking typically peaks during emerging adulthood (Ashenhurst et al., 2015; Chassin et al., 2002; Delucchi et al., 2008), a developmental period spanning ages 18–25 years. A variety of constraints that limit drinking ease during this developmental period (e.g., access to alcohol, parental monitoring, and peer normativity), and eases in each are linked to increases in emerging adult binge drinking (e.g., Carter et al., 2010; Sasser et al., 2023; White & Jackson, 2004). Initial increases in binge drinking during this period are followed by subsequent decreases, a pattern researchers refer to as the “maturing out” phenomenon (e.g., Lee & Sher, 2018). However, sustained high-risk drinking throughout emerging adulthood may disrupt this natural maturing process (e.g., Chassin et al., 2002; Lee et al., 2024; Waddell et al., 2022), potentially leading to continued heavy drinking and the development of alcohol use disorders in adulthood. Therefore, understanding factors that influence higher risk drinking during emerging adulthood is key to improving personalized early intervention.

The use of cannabis during emerging adulthood is known to potentiate risk for problematic drinking in individuals who use alcohol (e.g., Gunn et al., 2022; Lee et al., 2022). Studies have shown that more frequent cannabis use is cross-sectionally and prospectively associated with heavier drinking and negative alcohol use consequences over time (e.g., Gunn et al., 2022; Guttmannova et al., 2021; Waddell, 2023; Wardell et al., 2018), and that growth in alcohol and cannabis use covaries with one another (Guttmannova et al., 2021; Tucker et al., 2021). However, studies investigating within-person changes in levels of alcohol and cannabis use across development are less common. Within-person increases in frequency of cannabis use have been associated with an increased number of drinks per drinking day (Gunn et al., 2018) and negative alcohol consequences in college students (Wardell et al., 2020), and increased frequency of alcohol use in community samples (Guttmannova et al., 2021). While informative, there are notable limitations that the current study seeks to fill.

First, existing research on correlated trajectories of alcohol and cannabis use has employed data from samples that lack developmental trends essential to create and expand theoretical models and targeted intervention efforts. Our understanding of alcohol and cannabis use relationships over time can be enhanced by utilizing data collected across developmental periods when use patterns actively change, such as year-to-year assessment throughout emerging adulthood (aged 18–25 years). For instance, Guttmannova et al. (2021) found that alcohol and cannabis use frequency trajectories were correlated across a span of 2 years during young adulthood. A critical next step would be to test whether similar growth trajectories exist across emerging adulthood when age-specific yearly increases in drinking commonly occur (e.g., Lee et al., 2024). Studies that have used an age-based approach have been limited in covering the full span of emerging adulthood (e.g., up to age 22 years; Tucker et al., 2021) and focused on alcohol use frequency rather than binge drinking frequency. Thus, using an index of higher risk levels of drinking across ages 18–25 years would build upon these studies to characterize how cannabis use and frequency of binge drinking interrelate.

Second, it remains unknown whether age-specific within-person deviations in cannabis use predict subsequent within-person changes in binge drinking frequency 1 year later or vice versa. While it is well established from longitudinal and ecological momentary assessment research that increased cannabis use covaries with increased drinking frequency (e.g., Gunn et al., 2022; Guttmannova et al., 2021), it is unclear which comes first. Within-person research to date consistently shows that cannabis use potentiates levels of alcohol use; however, less clear is if levels of alcohol use potentiate levels of cannabis use (e.g., Gunn et al., 2022; Subbaraman, 2016; Waddell, 2021). Knowing whether within-person increases in cannabis use frequency potentiates binge drinking frequency (or vice versa) at specific ages would have direct implications for age-specific, developmentally tailored personalized interventions.

The current study sought to overcome these limitations by using annual developmental data on changes in cannabis use frequency and binge drinking frequency from ages 18–25 years. Utilizing parallel-process state–trait mixed effect growth modeling, the current study tested whether: (1) between-person levels of cannabis use frequency correlated with binge drinking frequency across emerging adulthood, (2) between-person growth in cannabis use frequency correlated with growth in binge drinking frequency across emerging adulthood, and (3) age-specific within-person changes in cannabis use frequency predicted subsequent increased binge drinking frequency and vice versa. We hypothesized that between-person levels and growth in frequency of cannabis use and binge drinking would be correlated, consistent with previous studies showing that alcohol and cannabis trajectories are correlated across various age periods and time spans (e.g., Guttmannova et al., 2021; Tucker et al., 2021). It was also hypothesized that within-person changes in frequency of cannabis use would predict subsequent changes in frequency of binge drinking, but not vice versa. This hypothesis was drawn from prior studies showing that co-use/use of the other substance potentiates risk for problematic drinking but not necessarily riskier cannabis use (e.g., Waddell, 2021). Differences by age within the emerging adulthood period were explored.

## METHODS

### Participants and procedures

Participants (*N* = 526; 50.3% female) were from the National Consortium on Alcohol and Neurodevelopment in Adolescence (NCANDA; Brown et al., 2015), a longitudinal study on the development and persistence of substance use. NCANDA uses a longitudinal accelerated cohort design to assess participants recruited at ages 12–21 years in 2013–2014 and followed annually for over 9 years. Participants were recruited at five sites: University of California, San Diego; SRI International in Menlo Park, CA; Duke University Medical Center in Durham, NC; University of Pittsburgh, PA; and Oregon Health & Science University in Portland, OR. A baseline assessment was followed by annual follow-ups. The current study used Data Release 9.0 (NCANDA_RELEASE_9Y_ REDCAP_MEASUREMENTS_V01), with participant data from baseline up to the 9-year follow-up. Participants provided assent (age 12–17 years) or consent (ages 18+ years) prior to each annual assessment. All study procedures were approved by the Institutional Review Board (IRB) of each site.

For the larger study (*N* = 831), participants were recruited into the study in two groups: a no/low substance use group in which most participants had no history of heavy drinking and little exposure to alcohol and other drugs when entering the study (*N* = 692), and an exceeds/substance using group where participants were allowed to have substantial experience with substance use (*N* = 139). Recruiting these two groups facilitated testing distinct longitudinal trajectories of substance use (Brown et al., 2015). Exclusion criteria in both groups at baseline were as follows: current diagnosis of severe psychiatric disorders (e.g., schizophrenia, bipolar disorder), substance dependence, current use of psychoactive medication, serious medical problems prohibiting completion of the protocol, intellectual disability or pervasive developmental disorder, lack of fluent English, uncorrected sensory impairment, and known prenatal drug or alcohol exposure (see Brown et al., 2015 for more details).

Participant requirements for the current study (*N* = 526 out of *N* = 831) were (1) having valid data during at least one time point from the age range of 18–25 and (2) reporting using alcohol and cannabis at least once during this age range. Retained participants had an average of 6.00 (SD = 2.39) data points during this age range. Participants in this subsample were 64% White/Non-Hispanic/Latinx, 12% African American/Black, 11% Hispanic/Latinx, 8% Asian, 4% Bi-racial/multiracial, 1% Pacific Islander, and 1% Native American/Alaskan Native (see Table 1).

### Measures

#### Past-year alcohol and cannabis use

Participants reported their past-year substance use via the Customary Drinking and Drug Use Record (CDDR; Brown et al., 1998), an interview-based assessment of alcohol, tobacco, and drug involvement assessed at each timepoint. The CDDR assessed past-year binge drinking by asking, “During the past year, how many times have you consumed 4 + (females)/5+ (males) drinks within an occasion?” The CDDR assessed past-year cannabis use frequency by asking, “In the past year, how many days did you use marijuana?” Participants reported a value between 0 and 365 for each question. Detailed “days of use” questions were skipped if participants report no substance use on a binary “yes/no” screening question at a given timepoint, and thus values of zero were imputed for these timepoints. The CDDR has been used across decades of research with adolescents and young adults, and has good test–retest reliability (e.g., Brown et al., 1998; Levy et al., 2004; Martin et al., 2000).

To reduce the large variances in binge drinking and cannabis use frequency, each outcome was divided by 12 (i.e., 12 months in 365 days), which allowed models to run seamlessly. As such, cannabis use frequency and binge drinking frequency are interpreted as average times per month rather than per year.

#### Demographics

Sex at birth and date of birth were collected at the baseline interview; thus, age was computed as a continuous age variable based upon the date of each assessment. SES was measured in terms of parental education level to avoid bias from significant regional income differences across sites.

### Data analytic plan

NCANDA’s accelerated longitudinal cohort design provides a wide range of ages at each assessment. Since each participant was enrolled at baseline and followed regardless of baseline age, missing data can be considered missing at random and suitable for multilevel models (see Baraldi & Enders, 2010). The “time” variable was derived from age data rather than year of assessment.

**FIGURE 1:**
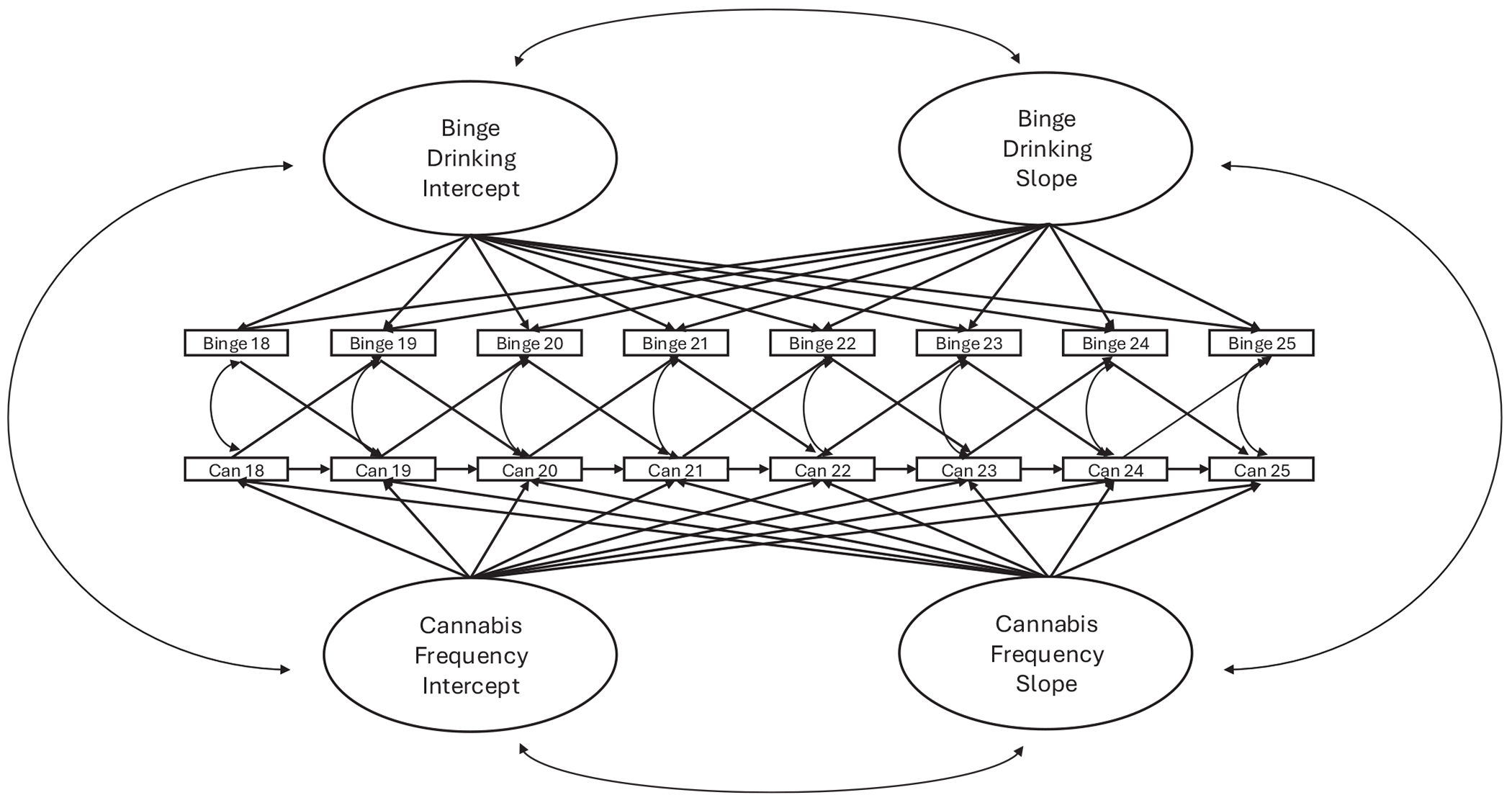
Conceptual model. “Can” stands for cannabis use frequency; “Binge” stands for binge drinking frequency, defined as the frequency of drinking 4+ drinks in a night for women, 5+ in a night for men.

Analyses consisted of state–trait mixed effect growth models (e.g., Geiser et al., 2015; Oeltjen et al., 2023; see supplemental material for statistical equations; Appendix S1). Parallel-process state–trait mixed effect growth models allow for dynamic growth processes across and within individuals, parsing stable between-person variability, between-person change, and dynamic within-person variability over time (see Figure 1). Within this model, random intercepts across people measure stable-like between-person variability, random slopes measure between-person change over time, and residual times-pecific variation measures leftover variability at a given time after factoring out an individual’s random intercept and slope function (i.e., residuals). The current study specified random intercepts as the stable, “trait-like” between-person variability, rather than as the “initial” levels at age 18 years, with the goal of testing both stable, trait-like between-person variance and rate of change/growth. Including two constructs, each with random intercepts, random slopes, and time-specific residuals, allowed the current study to estimate how each between-person and within-person component relate to one another over time (i.e., parallel processes).

In the parallel-process state–trait mixed effect growth model, we modeled random intercepts between binge drinking frequency and cannabis use frequency over time, as well as random slopes of binge drinking frequency and cannabis use frequency across time regressed on age (i.e., between-person growth). Correlations between the intercepts and slope functions of each parallel process were also specified to test whether higher frequency cannabis use across emerging adulthood was correlated with higher frequency binge drinking across emerging adulthood (i.e., stable, trait-like, and between-person effects), and whether steeper growth in cannabis use frequency across emerging adulthood was correlated with steeper growth in binge drinking frequency across emerging adulthood (i.e., dynamic and between-person change). Further, the model also tested (a) correlations between contemporaneous residual errors of binge drinking frequency and cannabis use frequency at a given age (e.g., covariance between age 18 residual error in binge drinking and age 18 residual error in cannabis use), (b) within-person carryover of binge drinking frequency and cannabis use frequency over time (e.g., residual of age 18 years binge drinking frequency predicting residual of age 19 years binge drinking frequency); (c) reciprocal prospective effects of the residuals of binge drinking frequency and cannabis use frequency (e.g., residual of age 18 years binge drinking frequency predicting age 19 years cannabis use frequency, and vice versa); and (d) sex at birth, socioeconomic status, site of data collection, and substance use group at baseline (1 = exceeds substance threshold, 0 = no/low substance use group) as between-person covariates of the random intercepts and slope functions. Finally, age interactions were included for the residual reciprocal effects to test whether relations between residual variation in binge drinking/cannabis use frequency at certain ages predicted residual variation in the other substance use frequency 1 year later. In the presence of a significant age interaction, simple slopes were estimated and compared across ages.

Empirical Bayesian estimation was used for analyses (e.g., Asparouhov & Muthén, 2010), and statistical significance was achieved if Bayesian credibility intervals (BCIs) did not contain zero (Eberly & Casella, 2003). Models were estimated in MPlus Version 8.9 using noninformative priors, specified by default in Mplus (Asparouhov & Muthén, 2010). Models were specified with 10,000 iterations to ensure model convergence, and the Potential Scale Reduction value was inspected, with optimal levels being under 1.05 (Gelman & Shalizi, 2013). Missing data were estimated under Bayesian estimation, using all observed data to estimate posterior distributions for parameters with missing data (Asparouhov & Muthén, 2023). Continuous predictors were centered based upon the level of analysis, with time-varying residuals centered around the person-mean and between-person variables centered around the sample mean (Enders & Tofighi, 2007; Hoffman & Stawski, 2009; Zhu et al., 2025). Binge drinking and cannabis use frequency were latent-mean centered (e.g., Asparouhov & Muthén, 2019; McNeish et al., 2023), which disaggregates within-person variance from between-person intercept and growth functions, and thus were specified using a standard Bayesian distribution. Skewness values were below 1.5 for both cannabis use (skew = 0.10) and binge drinking frequency (skew = 1.46). While some level of skewness is often present with substance use variables, Bayesian estimation does not assume a normal distribution and has been shown to be robust to nonnormal data patterns (Gelman & Shalizi, 2013).

Data were drawn from the ongoing NCANDA study (Baseline to Year 9 Data Releases of National Institute of Mental Health Data Archive Collection C4513). Collection and distribution of the NCANDA data were supported by NIH funding AA021681, AA021690, AA021691, AA021692, AA021695, AA021696, and AA021697. Access to NCANDA data is available via the NIMH Data Archive.

## RESULTS

### Descriptive statistics and intraclass correlations

Across timepoints, participants reported binge drinking 1.27 times per month (SD = 2.31) on average, and using cannabis 5.20 times per month (SD = 9.12) on average. A total of 50.7% (ICC = 0.493) of the variance in binge drinking frequency was attributable to within-person change over time; whereas 38.5% (ICC = 0.615) of the variance in cannabis use frequency was attributable to within-person change over time.

### State–trait mixed effects growth model

The potential scale reduction (PSR) value was 1.005 with 10,000 iterations, suggesting adequate convergence of the model. A full list of between-person model parameters can be found in Table 2. At the between-person level, the covariance between the random intercept of binge drinking frequency and cannabis use frequency was statistically significant ($\hat{\beta }=0.25$, SE = 0.08, 95% BCI = [0.10, 0.41]), suggesting that more frequent binge drinking across emerging adulthood was correlated with more frequent cannabis use across emerging adulthood. Further, the covariance between the random slope of binge drinking frequency and the random slope of cannabis use frequency was statistically significant ($\hat{\beta }=0.36$, SE = 0.16, 95% BCI = [0.01, 0.62]), suggesting that growth in binge drinking frequency across emerging adulthood was correlated with growth in cannabis use frequency across emerging adulthood. Covariances between the random intercept of binge drinking frequency and growth in binge drinking frequency ($\hat{\beta }=-0.30$, SE = 0.11, 95% BCI = [−0.48, −0.04]) and the random intercept of binge drinking frequency and growth in cannabis use frequency ($\hat{\beta }=-0.28$, SE = 0.09, 95% BCI = [−0.44, −0.07]) were also statistically significant, suggesting that individuals who engage in binge drinking more frequently across emerging adulthood reported less growth in binge drinking frequency and cannabis use frequency during emerging adulthood. Similarly, the random intercept of cannabis use frequency covaried with less growth in cannabis use frequency during emerging adulthood ($\hat{\beta }=-0.21$, SE = 0.08, 95% BCI = [−0.34, −0.04]) but did not covary with growth in binge drinking frequency ($\hat{\beta }=-0.05$, SE = 0.13, 95% BCI = [−0.31, 0.17]).

A full list of within-person model parameters is displayed in Table 3. At the within-person level, residual error in binge drinking frequency and cannabis use frequency covaried with one another at a given age ($\hat{\beta }=0.15$, SE = 0.02, 95% BCI [0.11, 0.19]), suggesting that residual within-person deviations in binge drinking frequency were accompanied by residual within-person deviations in cannabis use frequency. Residual within-person deviations in cannabis use frequency predicted subsequent residual within-person deviations in cannabis use frequency 1 year later ($\hat{\beta }=0.16$, SE = 0.03, 95% BCI = [0.10, 0.22]), whereas residual within-person deviations in binge drinking frequency did not predict residual within-person deviations in binge drinking 1 year later ($\hat{\beta }=0.01$, SE = 0.02, 95% BCI = [−0.04, 0.05]). When aggregated across ages, residual within-person deviations in binge drinking frequency did not predict subsequent residual within-person deviations in cannabis use frequency ($\hat{\beta }=0.01$, SE = 0.02, 95% BCI = [−0.04, 0.04]), and residual within-person deviations in cannabis use frequency did not predict subsequent residual within-person deviations in binge drinking frequency ($\hat{\beta }=0.02$, SE = 0.03, 95% BCI = [−0.04, 0.07]).

**FIGURE 2:**
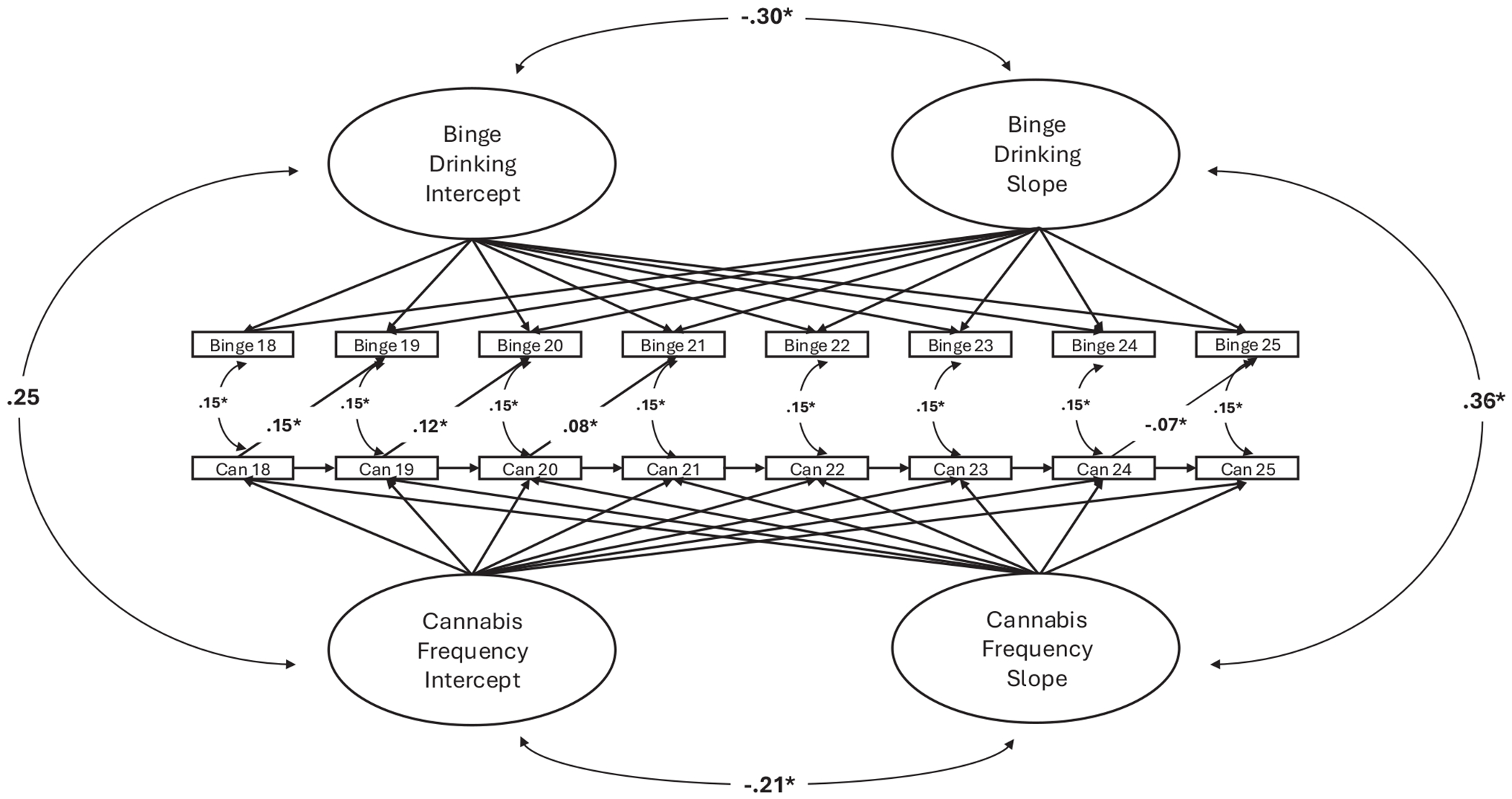
Parallel-process state–trait mixed effects growth model predicting within- and between-person relations between binge drinking and cannabis use. “Can” stands for cannabis use frequency at each age; “Binge” stands for binge drinking frequency (frequency of drinking 4+ drinks in a night for women, 5+ in a night for men) at each age. *95% Bayesian credibility intervals do not contain zero.

However, when age was included as a moderator, the interaction between age and residual within-person deviations in cannabis use frequency predicting subsequent residual within-person deviations in binge drinking frequency was statistically significant ($\hat{\beta }=-0.17$, SE = 0.05, 95% BCI = [−0.27, −0.06]). Simple slopes showed that residual within-person deviations in cannabis use frequency predicted subsequent residual within-person increases in binge drinking frequency at age 18 years ($\hat{\beta }=0.15$, SE = 0.06, 95% BCI = [0.05, 0.26]), age 19 years (*β* = 0.12, SE = 0.04, 95% BCI = [0.03, 0.20]), and age 20 years ($\hat{\beta }=-0.08$, SE = 0.03, 95% BCI = [0.01, 0.14]), did not predict residual within-person deviations at age 21 years ($\hat{\beta }=0.04$, SE = 0.03, 95% BCI = [−0.01, 0.09]), age 22 years ($\hat{\beta }=0.001$, SE = 0.03, 95% BCI = [−0.05, 0.05]), or age 23 years ($\hat{\beta }=-0.04$, SE = 0.03, 95% BCI = [−0.10, 0.02]), but predicted residual within-person decreases in binge drinking at age 24 years ($\hat{\beta }=-0.07$, SE = 0.04, 95% BCI = [−0.15, −0.01]; see Figure 2). The interaction between age and residual within-person deviations in binge drinking frequency predicting subsequent residual within-person deviations in cannabis use frequency was not statistically significant ($\hat{\beta }=-0.11$, SE = 0.05, 95% BCI = [−0.21, 0.01]).

In terms of covariates, male sex was associated with higher intercepts for binge drinking frequency ($\hat{\beta }=0.18$, SE = 0.06, 95% BCI = [0.07, 0.29]) and cannabis use frequency ($\hat{\beta }=0.18$, SE = 0.05, 95% BCI = [0.09, 0.28]) The other covariate effects of sex were not significant. Also not significant were socioeconomic status, site of data collection, and no/low versus higher drinking group (see Table 3).

### Sensitivity analyses

Although inclusion in the current study was for individuals who reported any alcohol and cannabis use at a given time between ages 18 and 25 years, *N* = 33 of the *N* = 526 individuals did not report a binge drinking episode. Thus, analyses were re-estimated with *N* = 493 individuals who reported both >1 cannabis use and a binge drinking episode throughout ages 18–25 years. Results were unchanged.

Outliers in binge drinking frequency (+3 SD from the mean) were present in 2.1% of cases. Analyses were re-estimated omitting these outliers; results were unchanged.

Nonlinear change and interactions are possible when modeling changes in binge drinking and cannabis use. Thus, models were re-estimated with quadratic random slope terms added to the models. When adding quadratic random slope parameters for binge drinking and cannabis use frequency, the only statistically significant correlation was between the quadratic and linear growth functions for cannabis use ($\hat{\beta }=-0.92$, SE = 0.02, 95% BCI = [−0.95, −0.87]) and quadratic and linear growth functions for binge drinking ($\hat{\beta }=-0.97$, SE = 0.01, 95% BCI = [−0.98, −0.95]). Furthermore, when specifying the cross-lagged residual effects with a quadratic interaction term, the interaction between residual within-person deviations in cannabis use frequency and age ($\hat{\beta }=-0.05$, SE = 0.04, 95% BCI = [−0.11, 0.05]) did not predict subsequent residual within-person deviations in binge drinking, nor did the quadratic interaction term between residual within-person deviations in binge drinking frequency and age predict subsequent residual within-person deviations in cannabis use ($\hat{\beta }=0.01$, SE = 0.04, 95% BCI = [−0.07, 0.08]).

## DISCUSSION

Despite growing interest in modeling the relations between alcohol use and cannabis use, vital gaps remain in our knowledge of how the two interrelate from a developmental standpoint, and how riskier binge drinking frequency, as opposed to frequency of any amount of alcohol, plays a role in developmental relations. The current study sought to fill several of these gaps by (1) including binge drinking frequency as an alcohol outcome instead of alcohol use frequency, (2) using data across emerging adulthood from ages 18–25 years, (3) testing whether between-person levels and escalation in binge drinking frequency are associated with levels and escalation in cannabis use frequency, and (4) testing whether dynamic, age-specific within-person changes in binge drinking frequency and cannabis use frequency prospectively predict changes in the other substance 1 year later. Findings indicated that between-person levels of binge drinking frequency and cannabis use frequency were correlated with one another, as were between-person growth functions in binge drinking frequency and cannabis use frequency. Furthermore, within-person residuals were contemporaneously correlated across ages, and within-person increases in cannabis use frequency predicted within-person increases in binge drinking frequency 1 year later from ages 18–22 years, but predicted within-person decreases in binge drinking frequency at ages 24–25 years.

At the between-person level, those who binge drank more frequently also reported more frequent cannabis use, and growth in binge drinking frequency correlated with growth in cannabis use frequency. This finding extends prior work looking at parallel trajectories (e.g., Guttmannova et al., 2021; Tucker et al., 2021), suggesting that correlated growth occurs through ages 18–25 years, particularly with binge drinking frequency as the alcohol outcome, rather than frequency of any level of drinking. As recommended drinking limits for young adults often focus more on quantity rather than frequency (e.g., Paula et al., 2020; National Institute on Alcohol Abuse and Alcoholism (NIAAA), 2025), findings may be particularly important in preventing high-risk relevant drinking in emerging adulthood. Findings show a pattern of complementarity (e.g., Subbaraman, 2016), wherein increases in cannabis use frequency are associated with increases in binge drinking frequency.

Findings also showed that within-person residual changes in cannabis use frequency and binge drinking frequency were correlated with one another at a given time point. Alternatively stated, within-person deviations in cannabis use frequency, which occurred outside of the normative growth function and between-person stable levels, were correlated with within-person deviations in binge drinking frequency also outside of the normative growth and stable levels of binge drinking frequency. These findings again show a pattern of complementarity (e.g., Subbaraman, 2016), now at the within-person level, wherein increases in cannabis use frequency and binge drinking frequency covary positively rather than diverge from one another.

Most interestingly, within-person residual increases in cannabis use frequency predicted subsequent within-person residual increases in binge drinking frequency during ages 18, 19, and 20years, with the largest slopes present at these earlier ages. This suggests that within-person changes in cannabis use frequency preceded within-person changes in binge drinking frequency from ages 18–21 years (see Figure 2). This pattern was not seen for within-person increases in binge drinking frequency predicting subsequent within-person increased cannabis use frequency. This may be because of major changes in environmental contingencies unfolding during these transition years (e.g., Patrick et al., 2018), or increases in cannabis use frequency indicate increased use for enhanced positive reinforcement (i.e., complementarity; Gunn et al., 2022), which then may motivate an individual’s continued binge drinking frequency over time. Prior studies suggest that using substances for positive reinforcement occurs in early stages of addiction development (e.g., Bekman et al., 2011), then transitions into negative reinforcement (i.e., coping) in later stages (e.g., Koob, 2013). Thus, given that prospective within-person effects of cannabis use frequency on binge drinking frequency were present early in emerging adulthood, findings may suggest that cannabis use frequency is a salient within-person predictor of risk for higher frequency binge drinking at the beginning stages of one’s drinking experiences.

Another possibility may be that role obligations (e.g., work, marriage, child rearing) in older emerging adults (e.g., ages 23–25 years) may be perceived to be inhibited by more frequent binge drinking (e.g., Lee & Sher, 2018). During these ages, an uptick in cannabis use frequency may not translate into increased binge drinking frequency. In fact, some individuals use cannabis for harm reduction (e.g., to reduce the effects of alcohol; Boyle et al., 2023), and increased cannabis use frequency during later emerging adulthood could indicate a switch away from higher risk drinking. This supposition is partially supported in that the simple slopes after age 23years became negative and became statistically significant at age 24 years. While speculative, additional research is needed to test how and why cannabis use frequency has age-varying prospective effects on within-person changes in binge drinking frequency.

Findings may have implications for preventive interventions. Given stable between-person relations between random intercepts and slope functions between cannabis use frequency and binge drinking frequency, interventions seeking to reduce binge drinking frequency may benefit from targeting cannabis use frequency, and interventions seeking to reduce cannabis use frequency may benefit from targeting binge drinking frequency. Furthermore, given that within-person residuals in cannabis use frequency predicted subsequent residuals in binge drinking frequency, monitoring fluctuations in cannabis use frequency and coupling increases in cannabis use frequency with adaptive prevention for binge drinking frequency may prevent future increases in binge drinking frequency. However, adaptive prevention may be most effective in the transition to emerging adulthood, ages 18–21 years, when prospective within-person relations were present.

Limitations include the focus on binge drinking frequency as an alcohol outcome, which is a step forward in understanding patterns of higher risk drinking in tandem with cannabis use over time, yet no similar proxy for “heavy” cannabis use frequency was incorporated (e.g., Prince et al., 2018). Future research could test between- and within-person relations between binge drinking frequency and indices of higher-potency/heavier cannabis use frequency, such as use of butane hash oil (BHO; Meier, 2017). Similarly, we do not know whether alcohol and cannabis use overlapped with one another (i.e., simultaneous or alternating use; Gunn et al., 2022), and future research on developmental trajectories of overlapping co-use is needed. Second, we did not include data after emerging adulthood and thus did not test how cannabis frequency influenced trajectories well into adulthood (e.g., after age 30 years), when normative decreases in use occur (e.g., Littlefield et al., 2012; Waddell et al., 2022). Future research should incorporate broader age ranges. Third, dynamic changes occurring on a more frequent basis (e.g., monthly or quarterly; Guttmannova et al., 2021) or in response to life changes were not examined, and future research using more frequent time intervals may be of particular value in untangling dynamics between binge drinking frequency and cannabis use frequency. Fourth, it is possible that early exposure to alcohol/cannabis use may affect developmental trajectories during adulthood, and future research should consider taking into account alcohol/cannabis use that occurs earlier in development. Finally, we did not consider the role that collegiate educational status may have played in study relations. While college occurs during the developmental period of young adulthood, there are many contextual characteristics to consider when considering the role of college (e.g., living arrangements, full-time versus part-time student status, membership in Greek life, financial aid/other work requirements). To give these factors associated with college adequate attention within the alcohol and cannabis co-use literature, future research investigating the comprehensive influences of these factors on changes in alcohol and cannabis use during young adulthood is needed.

Despite study limitations, the current study is the first to test within- and between-person dynamics between binge drinking frequency and cannabis use frequency using data capturing the full range of emerging adulthood. Individuals who binge drink more frequently also reported using cannabis more frequently, and the growth curves in both were correlated but not perfectly, implicating influences from variables untested herein. Within-person changes in cannabis use frequency predicted within-person increases in binge drinking frequency 1 year later during the beginning of emerging adulthood (aged 18–21 years), suggesting that therapeutic interventions targeting both alcohol and cannabis use frequency together may be especially effective early in adult development.

## Supplementary Material

Supplemental material

Additional supporting information can be found online in the Supporting Information section at the end of this article.

## Figures and Tables

**TABLE 1:** Sample characteristics.

	% or mean (standard deviation)
Demographics	
Sex at birth	50% female
Race/ethnicity	64% White/Non-Hispanic Latinx
	12% Black/African American
	11% Hispanic/Latinx
	8% Asian
	4% Bi-racial/Multiracial
	1% Pacific Islander
	1% Alaskan Native

Binge drinking frequency (per month)	
Age 18	0.57 (1.23)
Age 19	1.30 (2.30)
Age 20	1.48 (2.36)
Age 21	1.60 (2.75)
Age 22	1.66 (2.62)
Age 23	1.82 (3.27)
Age 24	1.23 (2.28)
Age 25	1.05 (1.74)

Cannabis use frequency (per month)	
Age 18	2.36 (5.95)
Age 19	4.04 (7.75)
Age 20	5.48 (8.98)
Age 21	6.57 (9.97)
Age 22	7.24 (10.53)
Age 23	7.21 (10.60)
Age 24	6.63 (10.66)
Age 25	5.86 (9.67)

*Note*: Binge drinking frequency and cannabis use frequency were measured as past-year days; however, due to the modeling approach, this number was divided by 12 to ascertain monthly averages. There were N = 452 observations at age 18, *N* = 453 at age 19, *N* = 452 at age 20, *N* = 442 at age 21, *N* = 416 at age 22, *N* = 332 at age 23, *N* = 271 at age 24, and *N* = 212 at age 25.

**TABLE 2:** Between-person parameters from the parallel-process state–trait mixed effects growth model of relations between binge drinking frequency and cannabis use frequency.

	$\hat{\beta }$	SE	95% Bayesian credibility intervals
Between-person effects			
**Binge drinking intercept ←→ Cannabis use intercept**	**0.25**	**0.08**	**(0.10, 0.41)**
**Binge drinking slope ←→ Cannabis use slope**	**0.36**	**0.16**	**(0.01, 0.62)**
**Binge drinking intercept ←→ Binge drinking slope**	**−0.30**	**0.11**	**(−0.48, −0.04)**
Cannabis use intercept **←→** Binge drinking slope	−0.05	0.13	(−0.31, 0.17)
**Binge drinking intercept ←→ Cannabis use slope**	**−0.28**	**0.09**	**(−0.44, −0.07)**
**Cannabis use intercept ←→ Cannabis use slope**	**−0.21**	**0.08**	**(−0.34, −0.04)**

Binge drinking intercept			
**Sex**	**0.18**	**0.06**	**(0.07, 0.29)**
SES (parental education)	−0.01	0.06	(−0.14, 0.11)
Site 2 vs. Site 1	−0.01	0.07	(−0.21, 0.08)
Site 3 vs. Site 1	0.02	0.08	(−0.19, 0.12)
Site 4 vs. Site 1	0.05	0.07	(−0.13, 0.16)
Site 5 vs. Site 1	−0.05	0.07	(−0.16, 0.13)
No/low drinking group	−0.06	0.06	(−0.19, 0.06)

Cannabis use intercept			
**Sex**	**0.18**	**0.05**	**(0.09, 0.28)**
SES (parental education)	−0.05	0.05	(−0.16, 0.05)
Site 2 vs. Site 1	−0.01	0.06	(−0.13, 0.11)
Site 3 vs. Site 1	0.02	0.06	(−0.11, 0.13)
Site 4 vs. Site 1	0.05	0.06	(−0.07, 0.17)
Site 5 vs. Site 1	0.06	0.06	(−0.07, 0.17)
No/low drinking group	−0.07	0.05	(−0.18, 0.03)

Binge drinking slope			
Sex	−0.12	0.08	(−0.28, 0.05)
SES (parental education)	−0.11	0.09	(−0.29, 0.05)
Site 2 vs. Site 1	0.02	0.11	(−0.16, 0.26)
Site 3 vs. Site 1	−0.17	0.10	(−0.37, 0.03)
Site 4 vs. Site 1	−0.14	0.10	(−0.34, 0.08)
Site 5 vs. Site 1	−0.04	0.10	(−0.24, 0.17)
No/low drinking group	0.13	0.08	(−0.04, 0.34)

Cannabis use slope			
Sex	−0.03	0.06	(−0.14, 0.09)
SES (parental education)	−0.01	0.06	(−0.14, 0.11)
Site 2 vs. Site 1	0.08	0.07	(−0.06, 0.21)
Site 3 vs. Site 1	0.01	0.08	(−0.14, 0.16)
Site 4 vs. Site 1	0.01	0.07	(−0.14, 0.15)
Site 5 vs. Site 1	0.01	0.08	(−0.17, 0.13)
No/low drinking group	−0.02	0.07	(−0.14, 0.11)

*Note*: The model incorporated data from ages 18–25 years in the NCANDA sample (*N* = 526); parameters are the between-person associations from the primary parallel-process state–trait mixed effect growth model displayed in Tables 2 and 3; statistically significant associations are bolded; binge drinking = binge drinking frequency, cannabis use = cannabis use frequency; bolded statistical parameters were statistically significant parameters.

**TABLE 3:** Within-person parameters from the parallel-process state–trait mixed effects growth model of relations between binge drinking frequency and cannabis use frequency.

	$\hat{\beta }$	SE	95% Bayesian credibility intervals
Within-person effects across age			
**T1 binge drinking ←→ T1 cannabis use**	**0.15**	**0.02**	**(0.11, 0.19)**
Binge drinking (T2)			
Binge drinking (T1)	0.01	0.02	(−0.04, 0.05)
Cannabis use (T1)	0.02	0.03	(−0.04, 0.07)
Age (T1)	0.04	0.03	(−0.02, 0.08)
Cannabis use (T2)			
**Cannabis use (T1)**	**0.16**	**0.03**	**(0.10,0.22)**
Binge drinking (T1)	0.01	0.02	(−0.04,0.04)
**Age (T1)**	**0.20**	**0.03**	**(0.14,0.24)**

Within-person interactions by age			
Binge drinking (T2)			
Cannabis use*age (T1)	**−0.17**	**0.05**	**(−0.27, −0.06)**
**Age 18 slopes**	**0.15**	**0.06**	**(0.05, 0.26)**
**Age 19 slopes**	**0.12**	**0.04**	**(0.03, 0.20)**
**Age 20 slopes**	**0.08**	**0.03**	**(0.01, 0.14)**
Age 21 slopes	0.04	0.03	(−0.01, 0.09)
Age 22 slopes	0.001	0.03	(−0.05, 0.05)
Age 23 slopes	−0.04	0.03	(−0.10, 0.02)
**Age 24 slopes**	**−0.07**	**0.04**	**(−0.15, −0.01)**
Cannabis use (T2)			
Binge drinking*age (T1)	−0.11	0.05	(−0.21, 0.01)

*Note*: The model incorporated data from ages 18–25 in the NCANDA sample (*N* = 526); parameters are the within-person associations from the primary parallel-process state–trait mixed effect growth model displayed in Tables 2 and 3; statistically significant associations are bolded; binge drinking = binge drinking frequency, cannabis use = cannabis use frequency; the top portion of the table comes from a first model without age interactions, whereas the bottom portion of the table comes from a second model with an age interaction; T1 refers to the age timepoint at a given observation, whereas T2 refers to the timepoint 1 year later (e.g., at age 18, T1 = age 18, T2 = age 19); bolded statistical parameters were statistically significant parameters.

## Data Availability

The data that support the findings of this study are openly available in National Institute of Mental Health Data Archive Collection at https://nda.nih.gov/, reference number C4513.
