# Supplementary material for: Mutual age-varying influences of binge drinking and cannabis use during emerging adulthood in the NCANDA cohort
Source: Alcohol Clin Exp Res (Hoboken). Author manuscript; Available in PMC 2026 Apr 7. (PMC13055478; doi:10.1111/acer.70139)
Supplement: Supplemental material [file NIHMS2152065-supplement-Supplemental_material.docx]

**Supplemental Material:**

**Parallel Process Mixed Effects Growth Model Formulas:**

Fixed and Random Effects Equation:

U_Alc_(j,t) = I_Alc_(j)+ S_Alc_(j) _*_t + ϵ_Alc_(t) + Covariates

U_Can_(j,t) = I_Can_(j) + S_Can_(j) _*_t + ϵ_Can_(t) + Covariates

Residual Effects Equation:

ϵ_Alc_(t2) = *a*_*_ϵ_Alc_(t1) + *b*_*_ϵ_Can_(t1) + *c*_*_(t1) + *d*_*_ϵ_Can_(t1)_*_(t1)

ϵ_Can_(t2) = *a1*_*_ϵ_Can_(t1) + *b1*_*_ϵ_Alc_(t1) + *c1*_*_(t1) + *d1*_*_ϵ_Alc_(t1)_*_(t1)

Definitions:

U_Alc_(j,t) = frequency of binge drinking at age ‘t’ for person ‘j’

U_Can_(j,t) = frequency of binge drinking at age ‘t’ for person ‘j’

I_Alc_(j) = Intercept of binge drinking frequency for person ‘j’

I_Can_(j) = Intercept of cannabis use frequency for person ‘j’

S_Alc_(j) = Slope of binge drinking frequency for person ‘j’

S_Can_(j) = Slope of cannabis use frequency for person ‘j’

ϵ_Alc_ (t) = random residual for binge drinking frequency at time ‘t’

ϵ_Can_(t) = random residual for cannabis use frequency at time ‘t’

ϵ_Alc_ (0) = random residual for binge drinking at age 18

ϵ_Can_(0) = random residual for cannabis use at age 18

t = age – 18 (i.e., t1 starts at 0).

a = residual of binge drinking frequency at age ‘t1’

b = residual of cannabis use frequency at age ‘t1’

*c =* age main effect on binge drinking frequency

*d* = interaction between age and residual of cannabis use frequency at age ‘t1’

a1 = residual of binge drinking frequency at age ‘t1’

b1 = residual of cannabis use frequency at age ‘t1’

*c1* = age main effect on cannabis use frequency

*d1* = interaction between age and residual of binge drinking frequency at age ‘t1’
